# A Retrospective Analysis of Cardiac Anatomy in Patients Undergoing the Fontan Operation

**DOI:** 10.7759/cureus.111903

**Published:** 2026-07-01

**Authors:** Sachin Talwar, Sandeepon Sarkar, Saurabh Gupta, Vishal V Bhende

**Affiliations:** 1 Cardiothoracic and Vascular Surgery, All India Institute of Medical Sciences, New Delhi, New Delhi, IND; 2 Cardiothoracic and Vascular Surgery, Command Hospital Air Force, Bengaluru, IND; 3 Cardiology, All India Institute of Medical Sciences, New Delhi, New Delhi, IND; 4 Pediatric Cardiac Surgery, Bhanubhai and Madhuben Patel Cardiac Centre, Shree Krishna Hospital, Bhaikaka University, Karamsad, IND; 5 Pediatric Cardiac Surgery, Sri Sathya Sai Sanjeevani Centre for Child Heart Care and Training in Pediatric Cardiac Skills, Kharghar, IND

**Keywords:** anatomy of single ventricle, cardiac morphology, fontan operation, single ventricle, univentricular heart

## Abstract

Background

Hearts suitable for Fontan palliation include those with an anatomically single ventricle (absence of a second ventricular mass), those with a single atrioventricular connection, and those with two ventricles in whom biventricular repair is not feasible due to anatomical or physiological complexity (functionally univentricular physiology). We describe the anatomical spectrum of such hearts over a decade at a single tertiary center.

Methodology

We encountered 420 patients undergoing the Fontan operation over a decade, between 2010 and 2019. Their detailed medical records were reviewed in this retrospective study, and the information gathered is presented in this article. The cardiac anatomy is presented depending on the information gathered from the medical records.

Results

A total of 420 patients underwent the Fontan operation during the study period. Only 30 (7.1%) had a true anatomically single ventricle. The remaining 390 had two ventricular chambers, including those with a hypoplastic ventricle. The most common anatomical diagnosis was double-outlet right ventricle (DORV) in 143 (34%) patients, followed by tricuspid atresia in 118 (28%) patients. Major aortopulmonary collateral arteries were present in 250 (59.5%) patients, and left superior vena cava in 61 (14.5%) patients. Completion Fontan operation after bidirectional Glenn shunt was the most common surgical pathway (n = 254, 60.5%), though primary Fontan (n = 15, 3.6%) and other staged approaches were also performed.

Conclusions

The anatomical spectrum of patients undergoing Fontan operation is wide and includes true anatomically single ventricle, single atrioventricular connection, and functionally univentricular physiology with two ventricular chambers. DORV was the most common diagnosis, followed by tricuspid atresia. The decision for Fontan operation was driven by a combination of anatomical and physiological factors. Early and long-term outcomes were not analyzed in this study.

## Introduction

The anatomy of hearts with a single ventricle has great diversity, including hearts with an anatomical or physiological single ventricle. The condition ranges from hearts with anatomically one ventricle, with types including hypoplastic right ventricle, tricuspid atresia, double-outlet right ventricle (DORV) with non-routable ventricular septal defect (VSD), double-inlet left ventricle (DIRV), and discordant atrioventricular connections with balanced ventricles. The concept of staging of the Fontan has gained popularity [1,2]. A variety of diagnoses deemed fit for the Fontan operation have emerged, and even patients with complex anatomical diagnoses, which have a higher surgical risk for anatomical biventricular correction, undergo the Fontan operation [3].

In the last decade, with the introduction of staged surgery with the final step being the Fontan operation, which has drastically decreased mortality, we are now able to incorporate even sicker children and provide them a better chance to live [2]. This has added more diagnoses to the list of patients who can undergo the Fontan operation. This has occurred due to improved understanding of pathophysiology, more surgical experience, and improved multidisciplinary management and staging surgeries, culminating in the Fontan operation. This has helped us to venture into territories of treating more anatomically complex hearts and undertake surgery previously labelled as a surgical intractability.

There has been a paradigm shift in the understanding of anatomical features and complexity in the cardiac anatomy of patients who can undergo the Fontan operation. This study aims to describe the anatomical spectrum and clinical/anatomical indications for the Fontan operation in patients treated at a single tertiary center over a 10-year period (2010-2019).

## Materials and methods

Study design and setting

This was a retrospective observational study conducted in the Department of Cardiothoracic and Vascular Surgery, All India Institute of Medical Sciences, New Delhi, India. The study included patients who underwent the Fontan operation at our institution over a 10-year period from January 2010 to December 2019. The study was designed to analyze the anatomical spectrum of patients undergoing the Fontan operation, with particular emphasis on differentiating patients with an anatomically single ventricle from those with two ventricles but functionally univentricular physiology or anatomical complexity precluding biventricular repair.

Ethical approval

The study protocol was approved by the Institutional Ethics Committee of All India Institute of Medical Sciences, New Delhi, India (approval number IECPG 369/23.06.2021, dated June 25, 2021). As this was a retrospective review of hospital records, individual informed consent was waived by the ethics committee. Patient confidentiality was maintained throughout data collection and analysis.

Study population

All patients who underwent the Fontan operation at our institution between January 2010 and December 2019 were screened for inclusion. A total of 420 patients were included in the final analysis. Patients were included if they had undergone either a primary Fontan operation or a completion Fontan operation during the study period and had adequate preoperative, intraoperative, and imaging records available for anatomical assessment. Patients with incomplete medical records or insufficient anatomical documentation were excluded from detailed subgroup analysis. All 420 patients had adequate records for inclusion in the main anatomical analysis. Cardiac catheterization data were available for 310 of 420 patients; the remaining 110 either had adequate non-invasive data or underwent primary Fontan without formal pre-Fontan catheterization.

Data sources and data collection

Data were obtained from hospital medical records, operative notes, echocardiography reports, computed tomography (CT) angiography reports, cardiac catheterization records, and intraoperative findings. The collected data were entered into a structured database for analysis. The following demographic and clinical variables were recorded: age at Fontan operation, sex, body weight, presence of cyanosis, room-air oxygen saturation, previous palliative procedures, and the type of Fontan operation performed.

The anatomical variables studied included atrial arrangement, atrioventricular connection, atrioventricular valve morphology, ventricular morphology, ventriculo-arterial connection, VSD morphology, pulmonary outflow obstruction, systemic and pulmonary venous abnormalities, presence of major aortopulmonary collateral arteries (MAPCAs), left superior vena cava, heterotaxy, dextrocardia, mesocardia, criss-cross ventricular relationship, juxtaposed atrial appendages, and other associated cardiac or vascular anomalies.

Anatomical assessment

All patients underwent a detailed preoperative anatomical evaluation. Transthoracic echocardiography was used as the primary diagnostic modality for defining segmental cardiac anatomy, including atrial situs, atrioventricular connections, ventricular morphology, ventriculo-arterial connections, atrioventricular valve anatomy, VSDs, and pulmonary outflow tract obstruction.

CT angiography was performed to further delineate extracardiac anatomy, including the Glenn circuit, pulmonary artery anatomy, systemic and pulmonary venous drainage, presence of left superior vena cava, MAPCAs, total or partial anomalous pulmonary venous connection, coronary artery anatomy, and associated arch anomalies.

Cardiac catheterization was performed as part of the institutional pre-Fontan evaluation protocol. The catheterization study was used to assess the patency of the bidirectional Glenn circuit, pulmonary artery anatomy, Glenn pressure, pulmonary artery pressures, ventricular function when available, and the presence of significant MAPCAs. Collaterals considered hemodynamically significant were occluded before the Fontan operation when feasible.

Intraoperative findings were reviewed to corroborate the preoperative diagnosis. Particular attention was given to anatomical features that may be difficult to define completely on preoperative imaging, such as juxtaposed atrial appendages, atrioventricular valve morphology, ventricular relationship, and surgical feasibility of biventricular repair.

Definitions and classification

For this study, patients were classified according to morphological and physiological criteria. An anatomically single ventricle was defined as a heart with only one identifiable ventricular chamber, with the absence of a second ventricular mass. Hearts with a double-inlet ventricular connection were also analyzed separately according to ventricular morphology.

Physiological single ventricle was defined as a heart with two ventricular chambers in which biventricular repair was considered unsuitable because of ventricular hypoplasia, non-routable VSD, severe atrioventricular valve straddling, unbalanced atrioventricular septal defect (AVSD), complex ventriculo-arterial connections, or other anatomical factors precluding safe or durable biventricular correction.

A hypoplastic ventricle was defined based on echocardiographic, angiographic, CT, and intraoperative assessment, showing an underdeveloped ventricular chamber considered inadequate to support either systemic or pulmonary circulation as part of a biventricular repair.

A non-routable VSD was defined as a VSD whose position, size, relationship to the great arteries, or associated intracardiac anatomy made intraventricular rerouting unsuitable or technically prohibitive for biventricular repair.

MAPCAs were recorded when identified on CT angiography or cardiac catheterization. Left superior vena cava, anomalous pulmonary venous drainage, abnormal coronary anatomy, and arch anomalies were recorded when present.

Surgical procedures

Patients underwent the Fontan operation either as a primary procedure or as part of staged univentricular palliation. In most patients, the Fontan operation was performed following a previous bidirectional Glenn shunt. Some patients had undergone earlier systemic-to-pulmonary artery shunt, pulmonary artery banding, atrial septectomy, or combinations of staged procedures before Fontan completion. The indication for the Fontan operation was determined by the treating surgical team based on anatomical suitability, physiological assessment, previous palliation, pulmonary artery anatomy, Glenn circuit function, pulmonary artery pressure, systemic ventricular function, atrioventricular valve competence, and feasibility of biventricular repair.

Statistical analysis

Data were analysed using SPSS Statistics software version 20.0 (IBM Corp., Armonk, NY, USA). Continuous variables such as age and weight were summarized using mean with standard deviation or median with range, depending on data distribution. Categorical variables were expressed as frequencies and percentages. As the objective of the study was to describe the anatomical spectrum of patients undergoing the Fontan operation, the analysis was primarily descriptive. No formal comparative hypothesis testing was performed; therefore, p-values and test statistics are not reported.

## Results

Demographics

Overall, 289 (68.8%) patients were male, and the rest were female (131, 31.2%). Patients’ ages ranged from 3 years to 24 years. Patients who underwent the Fontan operation were aged more than five years (median = 11 years). The weight ranged from 6 kg to 38 kg (median = 15 kg).

Clinical features

Cyanosis was present in 315 (75%) patients at birth, and the rest of the patients had features of increased Qp. Overall, 294 (70%) patients who underwent the Fontan operation had oxygen saturation of less than 75% in room air.

Echocardiography findings

On echocardiography, 20 different types of anatomy were found. In many patients, the anatomical features overlapped, but for the ease of description, they are mentioned separately. For example, an anatomical single ventricle and an anatomical single ventricle along with pulmonary stenosis are described separately. Similarly, tricuspid atresia and tricuspid atresia with pulmonary stenosis have been described separately.

The most common anatomical diagnosis was DORV, present across multiple subcategories: DORV with VSD and pulmonary stenosis (n = 108), DORV with malpositioned great vessels (n = 12), congenitally corrected transposition of great arteries (cc-TGA) with DORV (n = 11), DORV with AVSD and pulmonary stenosis (n = 6), and DORV with tetralogy of Fallot (TOF) (n = 6), totalling 143 (34%) patients.

Overall, 30 (7%) patients had an anatomical single ventricle. Further, 35 (8%) patients with AVSD underwent the Fontan operation. Patients having ventriculo-arterial discordance who underwent Fontan operation were segregated into two broad anatomical groups, namely, dextro-transposition of great arteries (d-TGA) and cc-TGA. Both categories had patients who also had VSD and pulmonary stenosis as associated anomalies. Overall, 59 (14%) d-TGA patients underwent the Fontan operation, and 19 (4%) cc-TGA patients underwent the operation. Overall, 78 (18%) patients had ventriculo-arterial discordance.

A double-inlet left ventricle was found in 15 (3.6%) patients. A double-outlet left ventricle was noted in 14 (3.3%) patients. Mitral atresia was found in eight (1.9%) patients, of whom three also had tricuspid atresia (both tricuspid and mitral atresia) (Table 1).

**Table 1:** Anatomical categories in percentage. Each patient is assigned to one primary anatomical diagnosis only (mutually exclusive categories). DORV subcategories total 143 patients: DORV with VSD and PS (108) + DORV with malpositioned great vessels (12) + cc-TGA with DORV (11) + DORV with AVSD and PS (6) + DORV with TOF (6). d-TGA = dextro-transposition of great arteries; VSD = ventricular septal defect; PS = pulmonary stenosis; cc-TGA = congenitally corrected transposition of great arteries; DORV = double-outlet right ventricle; TA = tricuspid atresia; DILV = double-inlet left ventricle; AVSD = atrioventricular septal defect; TOF = tetralogy of Fallot; DOLV = double-outlet left ventricle; PA = pulmonary atresia; IVS = interventricular septum; DIRV = double-inlet right ventricle

Anatomical categories	Frequency	Percent
d-TGA with VSD, PS	58	13.8
cc-TGA with DORV	11	2.6
DORV, VSD, PS with malpositioned great vessels	12	2.9
TA	45	10.7
TA with VSD, PS	73	17.4
DORV, VSD, PS	108	25.7
Single ventricle	24	5.7
Single ventricle, PS	6	1.4
DILV, large-inlet VSD, severe PS	15	3.6
DORV, PS, with AVSD	6	1.4
DORV, TOF	6	1.4
d-TGA, VSD, pulmonary atresia	1	0.2
AVSD, severe PS	4	1.0
DOLV, VSD, PS	14	3.3
AVSD, DORV, mitral atresia	4	1.0
DORV with non-routable VSD	2	0.5
cc-TGA VSD, PS	3	0.7
Pulmonary atresia	8	1.9
PA with IVS	3	0.7
DIRV	4	1.0
Unbalanced AVSD	13	3.1
Total	420	100.0

The anatomical categories can broadly be segregated further into those having a single ventricle (anatomical or physiological, that is, a single ventriculo-arterial connection) and double ventricles (Table 2).

**Table 2:** Anatomical and physiological single ventricle categories. TA = tricuspid atresia; VSD = ventricular septal defect; PS = pulmonary stenosis; DILV = double-inlet left ventricle; AVSD = atrioventricular septal defect; DORV = double-outlet right ventricle; PA = pulmonary atresia; IVS = interventricular septum; d-TGA = dextro-transposition of great arteries; cc-TGA = congenitally corrected transposition of great arteries; TOF = tetralogy of Fallot; DIRV = double-inlet right ventricle

Parameters	N	%
Anatomical single ventricle		
TA	45	10.7
TA with VSD, PS	73	17.4
Single ventricle	24	5.7
Single ventricle, PS	6	1.4
DILV, large-inlet VSD, severe PS	15	3.6
AVSD, DORV, mitral atresia	4	1.0
Pulmonary atresia	8	1.9
PA with IVS	3	0.7
Physiological single ventricle		
DORV, VSD, PS	108	25.7
d-TGA with VSD, PS	58	13.8
cc-TGA with DORV	11	2.6
DORV, VSD, PS with malpositioned great vessels	12	2.9
DORV, TOF	6	1.4
d-TGA,VSD, pulmonary atresia	1	0.2
DORV PS with AVSD	6	1.4
Unbalanced AVSD	13	3.1
DIRV	4	1.0
cc-TGA VSD, PS	3	0.7

CT angiography

CT angiography in patients scheduled for the Fontan operation was performed to determine the anatomy of the bidirectional Glenn (BDG) circuit and its patency, presence of MAPCAs, and the left superior vena cava, and to evaluate the systemic and pulmonary venous drainage and heterotaxy syndromes. Around 248 (59%) patients had MAPCAs that needed to be occluded in the cardiac catheterization laboratory using coils. The left superior vena cava was found to be present in 61 (14.5%) patients, which can be considered a common association. All patients had a functional and patent BDG circuit and normal anatomy of pulmonary arteries. Total anomalous pulmonary venous connection (TAPVC) was seen in four (1%) patients. Most patients had normal coronary artery anatomy. Only five (1.2%) patients had abnormal coronary anatomy (Table 3).

**Table 3:** CT angiography findings. CT findings are not mutually exclusive; a patient may appear in more than one subcategory (e.g., MAPCAs and LSVC). The total MAPCA-positive count of 250 is derived by summing all MAPCA-positive rows (rows 1, 3, and 4). MAPCAs = major aorto-pulmonary collateral arteries; LSVC = left superior vena cava; PA = pulmonary artery; TAPVC = total anomalous pulmonary venous connection; MPGA = malposed great arteries; CT = computed tomography

#	CT angiography findings	N	%
1	MAPCA-positive, no LSVC, adequate PA/Glenn circuit, normal coronaries, adequate PA/Glenn circuit	239	56.0
2	No significant MAPCA, normal PA, no LSVC	98	23.3
3	MAPCA and LSVC present	61	14.5
3a	MPGA	1	0.2
3d	TAPVC	4	1.0
3e	Dextro-position of aorta	5	1
3f	Bovine arch	4	1
3g	Double aortic arch	3	0.7
4	MAPCA-positive, normal Glenn, normal pulmonary artery, abnormal coronaries	3	0.7
5	MAPCA-negative, abnormal coronary	2	0.5
	Total	420	100.0

Cardiac catheterization study

A cardiac catheterization study was performed as an institutional protocol before a patient was subjected to the Fontan operation. We analyzed the superior cavopulmonary anastomosis (BDG circuit) and pulmonary arterial pressures. We also studied whether the antegrade flow at the time of BDG was interrupted during the patient’s last surgery before undergoing the Fontan operation.

Cardiac catheterization was performed in 310 of 420 patients; the remaining 110 did not undergo formal catheterization as they had adequate non-invasive data or underwent primary Fontan. Of the 310 catheterized patients, 163 (52.6%) had antegrade flow open, and 145 (46.8%) had interrupted antegrade flow. Glenn pressure was less than 15 mmHg in 287 of 310 (92.6%) patients and greater than 15 mmHg in 23 (7.4%). High Glenn pressure was more common in patients with interrupted antegrade flow (21 vs. 2). All patients had a patent Glenn circuit and normal pulmonary artery anatomy. During catheterization, 250 (59.5% of the total cohort) patients had major aortopulmonary collaterals, which were occluded before the Fontan operation (Table 4).

**Table 4:** Cardiac catheterization study.

Cardiac catheterization study	Frequency	Percent
Antegrade flow open, Glenn pressure less than 15 mmHg	163	52.6
Antegrade flow interrupted, Glenn pressure less than 15 mmHg	124	40.0
Antegrade flow interrupted, Glenn pressure more than 15 mmHg	21	6.8
Antegrade flow open, Glenn pressure more than 15 mmHg	2	0.6
Total	310	100

Indications for Fontan operation

The most common indication for the Fontan operation was tricuspid atresia. Overall, 30 (7.1%) patients who underwent the Fontan operation had an anatomically single ventricle. In cases of DORV with VSD, pulmonary stenosis was the most common indication, followed by the presence of a non-routable VSD, and an indication due to the presence of a hypoplastic right ventricle or left ventricle (Table 5).

**Table 5:** Indications for Fontan operation. VSD = ventricular septal defect; cc-TGA = congenitally corrected transposition of great arteries; PS = pulmonary stenosis

Indication for Fontan	Frequency	Percent
Anatomically only single ventricle	30	7.1
Hypoplastic or non-functional one ventricle	37	8.8
Single ventricle physiology	351	83.6
Non-routable VSD	1	0.2
cc-TGA, VSD, PS, non-routable VSD	1	0.2
Total	420	100.0

Surgical procedure

Completion Fontan operation was the most common procedure performed after a previous systemic-to-pulmonary artery shunt in the form of systemic-to-pulmonary artery shunt (BT shunt) and BDG. Primary Fontan operation was performed in 15 (3.6%) patients. Another common procedure was BDG followed by completion Fontan operation. Fontan operation after pulmonary artery banding and atrial septectomy was performed in two (0.5%) patients, and Fontan after pulmonary artery banding only was performed in four (1%) cases (Table 6).

**Table 6:** Surgical procedure. PA = pulmonary artery

Surgical procedure	Frequency	Percent
Completion Fontan	254	60.5
Blalock-Taussig shunt followed by completion Fontan	11	2.6
Primary Fontan	15	3.6
Bi-directional Glenn followed by completion Fontan	132	31.4
Post-PA band Fontan	4	1.0
PA band, atrial septectomy, bidirectional Glenn, and completion Fontan	2	0.5
PA band, bidirectional Glenn, and Fontan	2	0.5
Total	420	100.0

Intraoperative findings

Intraoperative findings corroborated with echocardiographic findings with respect to anatomy. The finding of juxtaposed atrial appendages was made most of the time intraoperatively.

Atria

The anatomical right atrium was defined using echocardiography and a CT scan. Intraoperatively, it was identified by the presence of a broad, triangular appendage with rough trabeculae. On the other hand, the left atrium was defined by the presence of a finger-like appendage and a smooth inner cavity. The most common type of atrial situs was situs solitus in 396 (94.3%) of all patients, and common atrium was seen in seven (1.7%) cases. Atrial situs inversus was present in one patient. Atria with right and left isomerism were seen in only two patients each. Indeterminate anatomy was seen in two cases (Table 7).

**Table 7:** Atrial anatomy.

Anatomy of atria	Frequency	Percent
Atrial situs solitus	396	96.4
Common atrium	7	1.7
Atria with right isomerism	2	0.5
Atria with left isomerism	2	0.5
Atrial situs inversus	1	0.2
Indeterminate	2	0.5
Total	420	100.0

Atrioventricular and ventriculo-arterial connection

The most common type of atrioventricular connection was concordant connections to the right artery to the right ventricle and the left artery to the left ventricle, which was seen in 395 (94.1%) patients, and in the remaining patients (25, 5.9%), it was discordant. Discordant connection comprised patients with cc-TGA. Ventriculo-arterial connection was concordant in 351 (83%) patients, while in 69 (17%) patients, it was discordant.

Ventricular septal defect

Overall, 371 (88.3%) patients had VSD, and 49 (11.7%) had an intact ventricular septum. The most common type of VSD was a large subaortic peri-membranous VSD. Overall, 28 (7%) patients had a large subaortic VSD; 70 (26%) hearts had a small VSD. Further, 21 (5%) patients had multiple muscular VSDs, 24 (5%) patients had AVSD, and 10 (2%) patients had a subpulmonary VSD. Doubly committed VSD was seen in 14 patients (Table 8).

**Table 8:** Ventricular septal defect. VSD = ventricular septal defect; PS = pulmonary stenosis; cc-TGA = congenitally corrected transposition of great arteries; AVSD = atrioventricular septal defect

VSD	Frequency	Percent
Intact ventricular septum	49	12
Very large VSD	110	26.0
Small restrictive	70	16.2
Large subaortic VSD	28	6.7
Inlet VSD	15	3.6
Non-reroutable VSD	48	11.4
Doubly committed VSD	14	3.3
Multiple muscular VSD	20	2
Type 2 AVSD	10	2.4
Large ASD with VSD	7	1.7
Multiple VSDs	1	0.2
AVSD	24	5.7
Subpulmonary VSD	10	1
cc-TGA, VSD, PS with non-routable VSD	1	0.2
Total	420	100.0

Atrioventricular valves

Overall, 143 (34%) patients had tricuspid atresia, and 8 (1.9%) patients had mitral atresia, of whom three also had tricuspid atresia. Straddling of atrioventricular valves was seen in 12 (2.9%) patients. Mild atrioventricular valve regurgitation (AVVR) was present in one patient. A single atrioventricular valve was seen in 35 (8.3%) patients (Table 9).

**Table 9:** Atrioventricular valves. TA = tricuspid atresia; MA = mitral atresia; AVVR = atrioventricular valve regurgitation; AV = atrioventricular; TV = tricuspid valve; MV = mitral valve

Atrioventricular valve	Frequency	Percent
Normal AV valves	212	50.5
TA	143	34.0
MA	8	1.9
Both TA and MA	3	0.7
Poorly developed TV/MV	14	3.3
Single AV valve	35	8.3
AVVR	1	0.2
AV valves with straddling	12	0.7
Total	419	99.8

Type of ventricle

Overall, 115 (27.3%) patients had a hypoplastic right ventricle, and 23 (5.5%) patients had a hypoplastic left ventricle. Further, 30 patients had a single ventricle. Of these, 15 (3.5%) had a morphological left ventricle type, 5 (1.2%) had a morphological right ventricle type, and 10 (2.4%) were of indeterminate type (Table 10).

**Table 10:** Morphology of the ventricle. RV = right ventricle; LV = left ventricle

Morphology of the ventricle	Frequency	Percent
Normal ventricles in morphology and relationship	252	60.0
Hypoplastic RV	115	27.3
Hypoplastic LV	23	5.0
Single ventricle with morphological RV type	5	1
Single ventricle with morphological LV type	15	3.5
Single ventricle indeterminate type	10	2
Total	420	100.0

Additional anatomical features

**Figure 1:**
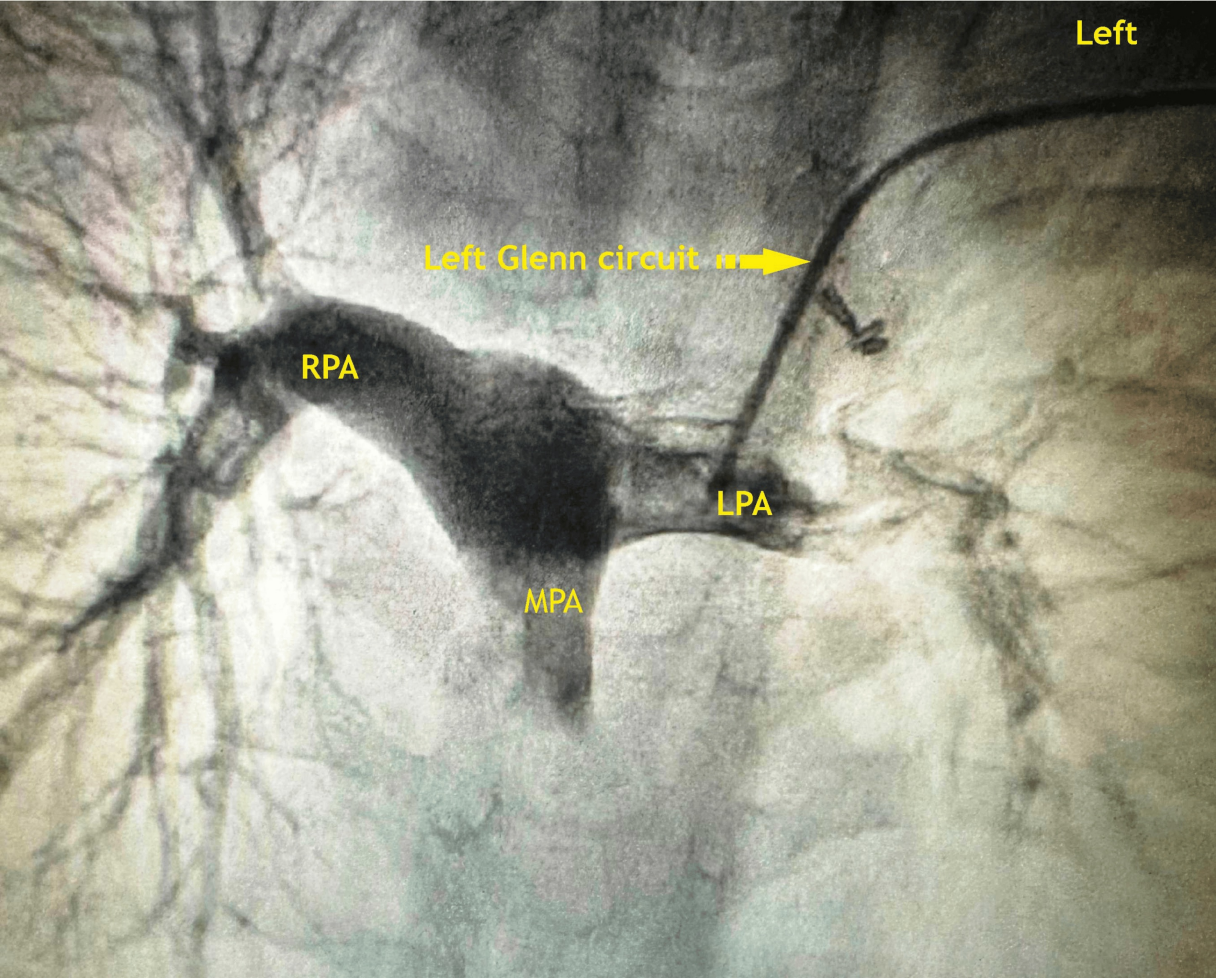
Angiography of the Glenn circuit. Selective pulmonary angiogram (anteroposterior projection) performed via the bidirectional Glenn circuit, demonstrating a patent Glenn anastomosis with opacification of the RPA and LPA. This is a representative image from the study cohort. RPA = right pulmonary artery; LPA = left pulmonary artery; MPA = main pulmonary artery Image credits: Dr. Sachin Talwar

The most common additional anatomical feature was dextrocardia in 14 (3.3%) patients. Heterotaxy was seen in eight (1.9%) patients, and left isomerism in two (0.5%) patients. Seven (1.7%) patients had Ebstein anomaly. Situs inversus with dextrocardia was seen in two (0.5%) patients. A criss-cross heart was seen in three (0.7%) patients. Mesocardia was present in 12 (2.9%) patients. Coarctation of the aorta was found in five (1.2%) patients, and a parachute mitral valve was noted in one (0.2%) patient (Table 11, Figure 1).

**Table 11:** Additional anatomical features. MS = mitral stenosis; COA = coarctation of aorta; PAPVC = partial anomalous pulmonary venous connection

Additional anatomical features	Frequency	Percent
No other anatomical abnormality except the main diagnosis	343	81.4
Dextrocardia	14	3.3
Heterotaxy	8	1.9
Left isomerism	2	0.5
Ambiguous situs	6	1.4
Crisscross heart	3	0.7
Ebstein’s anomaly	7	1.7
Situs inversus with dextrocardia	2	0.5
Parachute mitral valve with MS	1	0.2
PAPVC	3	0.7
COA	5	1.2
Juxtaposed appendages	4	1.0
Mesocardia	12	3
Situs inversus	8	2
Right isomerism	2	0.5
Total	420	100.0

## Discussion

Any heart subjected to the Fontan operation can be considered to have either an anatomically single ventricle, a physiologically single atrioventricular connection, or embody a series of complex anatomies precluding the possibility of biventricular repair. As the original procedure itself has undergone many modifications, there has been refinement in investigations and guidelines for choosing a specific anatomy for the Fontan operation. Detailed and accurate anatomical and physiological evaluation is required before subjecting these patients to the Fontan operation.

In this study, we collected data over a decade from 2010 to 2019. In total, 420 patients were analyzed. Of the 420 patients who underwent the Fontan operation, only 30 had an anatomically single ventricle; the remaining 390 had two ventricles, which included a hypoplastic ventricle as one of the two, and those who had both right and left ventricles. Decreased Qp was seen in 414 patients, and increased Qp was seen in 6 patients. In total, 330 patients undergoing the Fontan operation had an oxygen saturation ranging between 75% and 85% on room air.

Overall, 343 patients were aged over 5 years, and 340 patients weighed more than 10 kg. The mean age was 11 years. Anatomy was evaluated using echocardiography, CT angiogram, cardiac catheterization, and intraoperative findings. The anatomical features were grouped under the categories listed in Table 12.

**Table 12:** Anatomical categories observed in patients undergoing Fontan operation in the present study. AVSD = atrioventricular septal defect; cc-TGA = congenitally corrected transposition of the great arteries; DILV = double-inlet left ventricle; DIRV = double-inlet right ventricle; DOLV = double-outlet left ventricle; DORV = double-outlet right ventricle; d-TGA = dextro-transposition of the great arteries; PS = pulmonary stenosis; TOF = tetralogy of Fallot; VSD = ventricular septal defect

Anatomical category	Frequency, n	Percentage, %
d-TGA with VSD and PS	58	13.8
cc-TGA with DORV	11	2.6
DORV, VSD, PS with malpositioned great vessels	12	2.9
Tricuspid atresia	45	10.7
Tricuspid atresia with VSD and PS	73	17.4
DORV, VSD, and PS	108	25.7
Single ventricle	24	5.7
Single ventricle with PS	6	1.4
DILV with large inlet VSD and severe PS	15	3.6
DORV and PS with AVSD	6	1.4
DORV with TOF	6	1.4
d-TGA, VSD, and pulmonary atresia	1	0.2
AVSD with severe PS	4	1.0
DOLV, VSD, and PS	14	3.3
AVSD, DORV, and mitral atresia	4	1.0
DORV with non-routable VSD	2	0.5
cc-TGA, VSD, and PS	3	0.7
Pulmonary atresia	8	1.9
Pulmonary atresia with intact ventricular septum	3	0.7
Double-inlet right ventricle	4	1.0
Unbalanced AVSD	13	3.1
Total	420	100.0

DORV was the most common anatomical diagnosis, present in 143 of 420 patients across five subcategories (DORV with VSD and pulmonary stenosis, DORV with malpositioned great vessels, cc-TGA with DORV, DORV with AVSD and PS, and DORV with TOF). A double-outlet left ventricle was seen in 14 patients. Tricuspid atresia was the original indication for the Fontan procedure as devised by Fontan and Baudet [4]. In our series, tricuspid atresia was present in 118 patients. Mitral atresia was identified in eight patients, of whom three also had tricuspid atresia.

Disagreement exists over whether to call a heart with a single atrioventricular connection and a hypoplastic right or left ventricle a single ventricle or use the term single ventricle strictly for hearts with an actual ventricle only as a single ventricle [5-7]. Logically, a hypoplastic ventricle does not contribute to the functional aspect, but anatomists have been unyielding in ignoring their mere presence, even if they represent a small ventricular mass embedded in the systemic ventricle. In our series, 30 patients had a single ventricle in the absence of other ventricular masses in the heart. In 10 patients, it was of the indeterminate type. The left ventricular type was more common than the right ventricular type. In the series by Murari et al., the incidence of true single ventricle was three (1%) cases, and in the study by Barlow et al., it was five (5%) cases [8,9]. We agree with them about a true single ventricle being rare.

Discordant atrioventricular connection in patients undergoing the Fontan operation comprised both d-TGA and cc-TGA. These patients also had VSD and severe pulmonary stenosis. In total, 74 patients had d-TGA, and pulmonary atresia was also seen with pulmonary atresia in one case.

AVSD was another anatomical entity observed in 25 patients. They were seen in association with DORV or with severe pulmonary stenosis. Pulmonary atresia with non-routable VSD and pulmonary atresia with intact ventricular septum form another common anatomical group with 11 patients. DIRV was seen in four patients. The differences in our study from previously conducted studies were that it was a large experience of 420 over 10 years. In this study, we found DORV to be the most common anatomical anomaly in the hearts that underwent the Fontan operation.

In most patients, we found that the anatomical right atrium and left atrium exhibited situs solitus. A common atrium was seen in seven patients only. Atrial situs inversus was present in one patient. The variation in atria was not as common as the variation in ventricular anatomy. Juxtaposed atrial appendages were seen in four patients. In the series by Murari et al., there were nine patients, and Barlow et al. mentioned only three patients with isomeric atrial appendage and the rest with the usual atrial arrangement [8,9].

Normal right and left ventricles with a normal relationship were seen in 252 patients. Whereas a hypoplastic right ventricle was seen in 115 cases, and in 23 patients, it was a hypoplastic left ventricle. Anatomically single ventricle was seen in 30 patients. The anatomy of the solitary ventricle was of the left type in 15, indeterminate in 12, and the right ventricle type in three patients.

Overall, 34% of patients had tricuspid atresia, and around 2% of patients had mitral atresia. Straddling of atrioventricular valves was seen in 15% of patients. AVVR was seen in eight patients and was mild in nature. A single atrioventricular valve was seen in patients who had a single ventricle and in some patients with a hypoplastic second ventricle. Hypoplastic atrioventricular valves were seen in a single ventricle, as they could not be morphologically distinguished in any one type. Grade 3 straddling of valves and hypoplastic atrioventricular valves were also the determining factor for choosing Fontan over biventricular repair.

Comparison with other studies

There are many studies regarding the anatomy of hearts undergoing the Fontan operation. Studies by Murari et al. [8] and Barlow et al. [9] were published in 90s, two decades back. In the study by Murari et al., a total of 240 hearts were analyzed, and in the study by Barlow et al., 138 patients were analyzed. In our study, we analyzed the anatomy of 420 hearts with univentricular physiology. Akin to earlier studies, our methodology also involved echocardiography, cardiac CT angiography, cardiac catheterization study, and intraoperative findings (Table 13).

**Table 13:** Comparison with other studies. VA = ventriculo-arterial; DORV = double-outlet right ventricle; IVS = intact ventricular septum; AV = atrioventricular

Features	Murari et al. [8]	Barlow et al. [9]	Present study
Total patients	240	138	420
Univentricular connections	104	89	168
True single ventricle	3 (1%)	5 (3%)	30 (7%)
Biventricular connection	136	49	252
VA concordant/discordant	12/25	3/30	351/69
Hypoplastic ventricles	52	45	158
Balanced ventricles	86	49	252
DORV	76	7	134
Tricuspid atresia/Right AV valve atresia	80	26	118
Pulmonary atresia with IVS	1	12	11

There were no patients with hypoplastic left heart syndrome in our series, whereas Barlow et al. mentioned three patients with hypoplastic left heart syndrome. Left superior vena cava was encountered in 60 patients in our study. A criss-cross ventricle was observed in one case in both previous studies; in our study, it was noted in three patients.

Other additional anomalies noted in our study were TAPVC in two patients, partial anomalous pulmonary venous connection in three patients, mesocardia in 12 patients, dextrocardia in 14 patients, situs inversus in eight patients, and juxtaposition of the atria in four patients. Indeterminate-type single ventricle anatomy was seen in 10 patients. However, we did not find any patients having significant pulmonary artery bifurcation stenosis.

CT scan

In the CT scans, most patients who underwent the Fontan operation also had significant MAPCAs. They underwent occlusion of these MAPCAs using coils in the cardiac catheterization laboratory before the Fontan operation. The presence of the left superior vena cava was not uncommon and was seen in 60 (14%) patients. Abnormalities in the coronary arteries were uncommon and seen in only two (0.5%) patients. In both patients, there was no abnormality in the coronary artery origin. However, an abnormality was seen in the form of aneurysmal dilatation of the coronary arteries. Only four patients had a TAPVC.

Cardiac catheterization

Cardiac catheterization was performed to determine the anatomy of the BDG circuit and flow through it. It was also helpful in determining the pressure of BDG, which indirectly measures the pulmonary artery pressure and is essential before subjecting the patient to the Fontan operation. The majority of the patients who underwent the Fontan operation had BDG pressure less than 15 mmHg (n = 287). Antegrade flow interruption was also evaluated, and 164 patients had antegrade flow open, and 145 patients had antegrade flow interrupted at the time of BDG. Most patients who had significant MAPCA underwent occlusion of these in the cardiac catheterization laboratory before the Fontan operation. In the experience of Murari et al. and Barlow et al., not much emphasis was given to this aspect involving MAPCAs, Glenn anatomy, and Glenn pressure before FO (Table 14).

**Table 14:** Types of ventricular septal defects encountered in patients undergoing Fontan operation in the present study. ASD = atrial septal defect; AVSD = atrioventricular septal defect; cc-TGA = congenitally corrected transposition of the great arteries; PS = pulmonary stenosis; VSD = ventricular septal defect

Type of VSD	Frequency, n	Percentage, %
Intact ventricular septum	49	11.7
Very large VSD	110	26.2
Small restrictive VSD	70	16.7
Large subaortic VSD	28	6.7
Inlet VSD	15	3.6
Non-routable VSD	48	11.4
Doubly committed VSD	14	3.3
Multiple muscular VSDs	20	4.8
Type 2 AVSD	10	2.4
Large ASD with VSD	7	1.7
Multiple VSDs	1	0.2
AVSD	24	5.7
Subpulmonary VSD	10	2.4
cc-TGA, VSD, and PS with non-routable VSD	1	0.2
VSD morphology not specified/unclassified in available records	13	3.1
Total	420	100.0

VSD was present in most patients who underwent the Fontan operation. Overall, 370 (88%) had a VSD, and 49 (12%) had an intact ventricular septum. A very large VSD was the prominent feature in 110 patients, sometimes as large as leaving just a thin rim of septum and appearing as a single ventricular chamber. The diagnosis of non-routable VSD was more of an echocardiographic diagnosis, along with intraoperative findings, leading to termination into a univentricular palliative surgery instead of biventricular definitive repair. Multiple VSDs in the form of muscular VSDs and more than two defects, including perimembranous, muscular, and inlet VSDs, were also present in 21 patients. Patients with multiple VSDs underwent pulmonary artery banding before the Fontan operation. Large ASD, along with VSD, was seen in eight patients. AVSD was also seen in 21 patients.

Other additional morphological features were noticed in the form of dextrocardia (n = 14) and Ebstein anomaly (n = 7). Indications for the Fontan operation in patients with Ebstein anomaly were severe pulmonary stenosis and non-routable VSDs [10]. Six patients had a criss-cross heart, that is, the relationship between the ventricular chambers was superior-inferior. Dextrocardia and situs inversus were also seen in eight patients. Five patients had mesocardia. Coarctation of the aorta was found in two patients.

The decision of a Fontan operation

**Figure 2:**
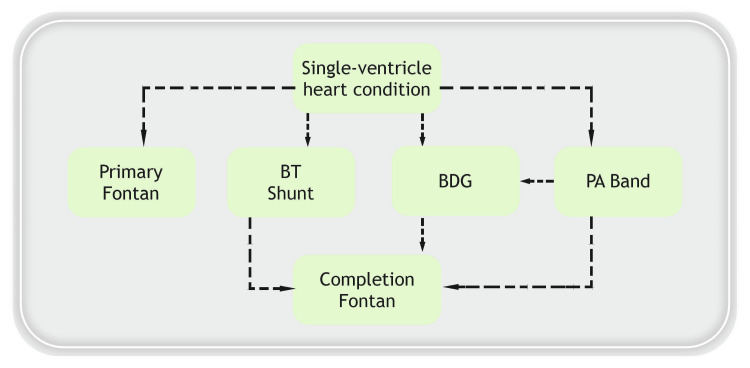
Surgical strategies in univentricular hearts. BT =  Blalock-Taussig; BDG = bidirectional Glenn shunt; PA = pulmonary artery Image credits: Dr. Vishal V. Bhende.

The understanding of the Fontan physiology has been evolving. A study by Hopkins et al. in 1985 described the significance of staging the Fontan procedure and emphasized the utility of BDG before the Fontan operation [11]. In 1992, Norwood and Jacobs suggested that performing a single-stage Fontan results in a sudden change in the hemodynamics and adversely alters the ventricular diastolic function [1,2]. They concluded that staging the Fontan operation improved outcomes. Norwood and Jacobs further presented a series of 100 consecutive staged FO without any deaths [1,2]. The concept of pre-optimization of a patient for a Fontan operation saw the daylight. This has allowed the utilization of the Fontan pathway for a larger patient population and the mid and long-term follow-up, given benefits in terms of survival and morbidity [12-14]. In our study, FO in most patients was offered in the form of completion Fontan, which was a part of staged management (Figure 2).

In patients with reduced pulmonary blood flow, the patients were offered a BT shunt on presentation in the neonatal period or infancy. At the age of 2-4 years, they underwent BDG, and later, when the age was more than five years, they underwent completion Fontan operation. Patients with excessive pulmonary blood flow underwent pulmonary artery banding as the first procedure, followed by BDG/Fontan operation. This strategy of staged Fontan operation was applied in most patients (n = 254, 60%) in our series; 132 patients underwent BDG followed by completion Fontan operation. In 11 patients, the Fontan operation was performed after a BT shunt only without an intermediate BDG. Primary Fontan operation was performed in 15 patients who had an anatomically single ventricle, who underwent Fontan operation either primarily or as a part of staged management, terminating in completion Fontan operation.

In patients with ventriculo-arterial discordance, that is, patients with d-TGA and cc-TGA with VSD, pulmonary stenosis was the most common reason for the selection of Fontan over biventricular repair, with an inability to route the VSD. Other reasons were the presence of complicated anatomy with smaller atria, a hypoplastic atrioventricular valve, dextrocardia, and patients with inlet VSD in cc-TGA, where a double-switch operation was not feasible. Patients with d-TGA with VSD and pulmonary stenosis underwent Fontan operation due to the presence of (a) Ebstein anomaly, (b) multiple VSDs, (c) borderline right ventricular function, (d) hypoplastic right ventricle, (e) hypoplastic ventricle, and (f) atrioventricular valve hypoplasia.

Patients with DORV, AVSD, and pulmonary stenosis also underwent Fontan operation instead of a biventricular correction, mainly due to complicated anatomy precluding a biventricular repair. These were unbalanced AVSD, a non-routable VSD, ventricular right or left ventricular hypoplasia, the presence of Ebstein anomaly with AVSD, and situs inversus along with dextrocardia.

There is a considerable heterogeneity in hearts undergoing the Fontan operation. According to the Anderson group, an anatomical single ventricle is a rarity. According to this group, using the term single atrioventricular connection defines a univentricular physiology more appropriately [6]. They believe that true single ventricles are a relatively uncommon entity on their own. In most patients, another ventricle is present, which is physiologically not sufficient to sustain the pulmonary or systemic circulation; this finding in our study remained consistent with other studies.

Assessment of the atrioventricular junction plays an important role in the decision-making to undergo a Fontan operation. In this study, we conclude that even patients who had both ventricles with both atrioventricular valves present underwent the Fontan operation due to the presence of more than one anatomical factors averting a biventricular repair. Presence of unbalanced ventricles, inability to route the VSD, straddling of atrioventricular valves, and co-existent Ebstein anomaly are some of the factors. Apart from anatomical premonition, a surgeon’s perspective also comes into play when decision-making has to be done intraoperatively. Often, a difficult decision is to choose between a very complex anatomical correction and a simpler Fontan operation. Typical examples are patients with cc-TGA with an unroutable/complex VSD, patients with a very large VSD or an unbalanced AVSD, or other factors requiring a complex correction. Fontan operation in these subsets is often preferred over a complex anatomical correction. Prior studies have reported comparable midterm results between Fontan palliation and complex biventricular repair in selected anatomical subgroups [15,16]. In patients with d-TGA with VSD and pulmonary stenosis, prior data suggest that Fontan may be associated with lower early mortality and lower reintervention rates compared with complex biventricular repair [17]. These conclusions are based on cited literature and were not analyzed in the present study.

Choussat’s criteria [18] are no longer the only factors on which indications for Fontan operation are based. Pulmonary artery pressure and function of the systemic ventricle are now significant factors considered before subjecting these patients to a Fontan operation. Thus, apart from anatomical factors, the physiologic factors obtained by echocardiography and cardiac catheterisation also have a role in the decision-making.

Limitations

This study has a few limitations. As a retrospective, single-center study, the findings depend on the completeness and accuracy of available medical records, imaging reports, catheterization data, and operative notes. Although the study included a large cohort of patients undergoing Fontan operation, it primarily described anatomical patterns and indications for Fontan palliation; early and long-term outcomes were not analyzed. Classification of complex cardiac anatomy may have some overlap, especially in patients with DORV, unbalanced AVSD, ventricular hypoplasia, straddling atrioventricular valves, non-routable VSD, heterotaxy, or ventriculo-arterial discordance. In addition, quantitative parameters such as ventricular volumes, atrioventricular valve dimensions, pulmonary vascular resistance, and detailed pulmonary artery indices were not uniformly available for all patients. Finally, the decision to proceed with the Fontan operation was influenced by institutional practice and surgical judgement, which may limit the generalizability of the findings.

## Conclusions

The anatomical spectrum of patients undergoing the Fontan operation is wide, encompassing true anatomically single ventricle, single atrioventricular connection, and functionally univentricular physiology with two ventricular chambers. DORV was the most common anatomical diagnosis, followed by tricuspid atresia. The selection of Fontan over biventricular repair was driven by a combination of anatomical factors, including non-routable VSD, ventricular hypoplasia, atrioventricular valve straddling, and complex ventriculo-arterial connections, and physiological parameters. Early and long-term outcomes were not analyzed in this study.
